# Extent of Coronary Stenosis and Anxiety Symptoms among Patients Undergoing Coronary Angiography

**Published:** 2017-10

**Authors:** Shervin Assari, Hassan Zandi, Khodabakhsh Ahmadi, Davoud Kazemi Saleh

**Affiliations:** 1 *Department of Psychiatry, University of Michigan, Ann Arbor, MI, USA.*; 2 *Center for Research on Ethnicity, Culture and Health, School of Public Health, University of Michigan, Ann Arbor, MI, USA. *; 3 *Medicine and Health Promotion Institute, Tehran, Iran.*; 4 *Behavioral Sciences Research Center, Baqiyatallah University of Medical Sciences, Tehran, Iran.*

**Keywords:** *Coronary artery disease*, *Coronary angiography*, *Anxiety*, *Coronary stenosis*

## Abstract

**Background:** The association between coronary angiographic findings and the level of anxiety symptoms among patients who undergo coronary angiography is not known. The aim of this study was to investigate the association between the extent of coronary stenosis and anxiety symptoms in patients who undergo coronary angiography.

**Methods:** In a cross-sectional study, 106 patients who underwent coronary angiography and had varying degrees of coronary artery disease were enrolled. Demographic characteristics (i.e., age and gender), socioeconomic status (i.e., educational attainment, income, and marital status), and traditional risk factors (i.e., hypertension, diabetes mellitus, hyperlipidemia, and smoking) were measured. The independent variable was the extent of coronary stenosis shown by coronary angiography, coded as single-vessel disease (n = 19), 2-vessel disease (n = 28), or 3-vessel disease (n = 59). The main outcome was symptoms of anxiety measured using the Hospital Anxiety Depression Scale (HADS). The Kruskal–Wallis test was used for bivariate analysis, and linear regression was applied for multivariable analysis.

**Results:** Participants were mostly men (n = 78, 73%), at a mean age of 50.14 ± 10.60 years. We found an inverse association between the extent of coronary stenosis and anxiety symptoms in our samples. Anxiety symptoms were lowest in the patients with 3-vessel disease and highest in those with single-vessel disease. The above association remained significant in a linear regression model, controlled for the demographic, socioeconomic, and traditional risk factors.

**Conclusion:** An inverse association may exist between the extent of coronary stenosis and the severity of anxiety symptoms in patients who undergo coronary angiography. Patients who undergo angiography and have fewer angiographic findings require screening for anxiety symptoms.

## Introduction

Due to an increase in industrialized lifestyles, the prevalence of coronary artery disease (CAD) has dramatically risen during the past few decades. Research has documented that patients with CAD have higher anxiety symptoms than controls.^1^ One study found a high level of anxiety symptoms among 80% of patients with CAD.^2^

Anxiety and CAD have a mutual relationship as the effect of long-lasting anxiety on atherosclerosis is well known^3^^-^^8^ and CAD, secondary to lifestyle modifications, can cause anxiety.^9^ CAD diagnostic procedures produce anxiety by themselves.^10^^, ^^11^ The worsening of patients’ quality of life, morbidity, mortality, and disease progression is among the vicious effects of anxiety in patients with CAD. The presence of anxiety in addition to these effects can lead to more regular visits and better assessments of patients’ compliance and health.^12^ Anxiety often worsens the prognosis in patients with CAD.^13^^-^^16^ Anxiety characteristics also independently and prospectively predict myocardial infarction.^17^

A systematic review of prospective studies showed evidence for the impact of anxiety on the etiology and prognosis of CAD.^18^ Another systematic review of 31 studies showed that after controlling for the effect of CAD severity, comorbid anxiety increased the number of CAD symptoms.^19^ Thus, evaluation of anxiety may optimize CAD management.^19^

Angiography is one of the standard diagnostic procedures for the evaluation of the severity of coronary artery stenosis in patients with CAD.^20^ The extent of coronary stenosis found in angiography predicts morbidity, mortality, and quality of life.^21^^, ^^22^

There are also studies reporting an association between personality traits and angiographic findings.^11^ Channer et al.^23^ showed that negative (normal) exercise test results were associated with higher anxiety symptoms in patients with CAD. There is, however, a paucity of studies assessing the association between the extent of coronary stenosis and anxiety symptoms.

We conducted this study to assess the association between the severity of coronary stenosis and symptoms of anxiety in patients who undergo coronary angiography.

## Methods

This cross-sectional study involved 106 patients who underwent coronary angiography for the first time in Tehran, Iran. Angiography was conducted at the request of a cardiologist. Demographic data (including age, gender, educational level, marital status, and place of residence) and CAD-related clinical characteristics (including history of hypertension, diabetes mellitus, hyperlipidemia, and cigarette smoking) were recorded.

Anxiety was assessed using the Hospital Anxiety Depression Scale (HADS) questionnaire. HADS consists of 7 questions (each rated 0–3) to evaluate anxiety. A HADS anxiety score ranges from 0 to 21, where a higher score reflects higher anxiety symptoms. Scores above 8 were considered presumptive anxiety symptoms, scores above 11 moderate anxiety, and scores equal to or greater than 16 severe anxiety symptoms.^24^ The HADS questionnaire is a routine modality for the evaluation of anxiety in different countries, and its usage in published articles quadrupled between 1996 and 2002.^25^ This questionnaire has previously been widely drawn upon to assess anxiety and depression in patients with CAD.^26^ Montazeri et al.^27^ validated this questionnaire for application in the Iranian population in 2003. The Cronbach’s alpha of this questionnaire was 0.771 for the anxiety score and 0.815 for the whole questionnaire.

The sample size needed to compare k means for 1-way ANOVA pairwise, 2-sided equality was calculated. The sample size was 106 considering the means of the groups to be 6 and 10 with an SD of 9 and the number of pairwise comparisons to be 3. The statistical analyses were done using SPSS for Windows, version 13 (*SPSS*, Inc., Chicago, IL, USA). The categorical and nominal variables were described using frequency tables. The normally distributed continuous variables, such as age, were reported as means ± SDs. Regarding the non-normal distribution of anxiety scores, documented by the one-sample Kolmogorov–Smirnov test, the total anxiety scores were reported as medians and 25th and 75th percentiles. The Kruskal–Wallis test was used to compare the anxiety scores between the different patient groups regarding their angiographic findings. A χ^2^ test was employed to compare the frequency of abnormal anxiety symptoms in the patient groups. The existence of the possible association between stenosis severity and anxiety symptoms was tested via a linear regression model in which the level of anxiety symptoms was the outcome. Our regression only included independent variables which were significantly associated with anxiety symptoms in our bivariate analysis. There was no evidence suggesting multicollinearity. A p value < 0.05 was considered significant. 

## Results

The range and mean (SD) of the age of the patients were 38 to 82 and 50.14 (10.6) years, respectively. 

Based on the coronary angiography findings, the patients had either a single-vessel disease (n = 19), 2-vessel disease (n = 28), or 3-vessel disease (n = 59). These groups were not significantly different vis-à-vis the demographic data such as age; gender; marital status; income; educational level; and history of hypertension, diabetes mellitus hyperlipidemia, and cigarette smoking (Table 1).

**Table 1:** Characteristics of the participants based on the severity of coronary stenosis in angiography*

	Single-vessel disease (n=19)	Two-vessel disease (n=28)	Three-vessel disease (n=59)	P value
Age (y)	48.07±5.42	50.88±5.56	50.46±6.09	0.424
Male	11 (57.9)	19 (67.8)	48 (81.4)	0.078
Married	15 (78.9)	25 (89.2)	54 (91.5)	0.451
Diploma and above	5 (26.3)	7 (25.0)	21 (36.2)	0.603
Living in rural areas	18 (94.7)	26 (92.8)	52 (88.1)	0.712
Monthly income > $200	13 (68.4)	19 (67.8)	38 (65.5)	0.939
History of diabetes	10(52.6)	14 (50.0)	31(52.5)	0.973
History of hypertension	5 (26.3)	7 (25.0)	17 (28.8)	0.927
History of hypercholesterolemia > 200 mg/dL	8 (42.1)	11 (39.2)	20 (33.8)	0.772
Smoking	6 (31.5)	9 (32.1)	14 (23.7)	0.643

* Data are presented as mean±SD or n (%).

For all the patients, the median and 25th to 75th percentile range of anxiety score were 5 and 3 to 9, respectively. 

The severity of anxiety symptoms was significantly different between the 3 patient groups. The lowest anxiety scores belonged to the patients with 3-vessel disease, and the highest scores belonged to those with single-vessel disease (Table 2).

The inverse association between the angiographic findings and the severity of anxiety symptoms, found in the bivariate analysis (using the Kruskal–Wallis test), was replicated in a linear regression model, which also controlled for all the covariates (i.e., age; gender; marital status; income; educational level; and history of hypertension, diabetes mellitus hyperlipidemia, and cigarette smoking).

**Table 2:** Comparison of anxiety symptoms based on the severity of coronary stenosis among the patients, who underwent coronary angiography*

Coronary Angiography Find- ings	Range	25th and 75th Percentiles	Median
Single-vessel disease (n=19)	1–21	4–17	8
Two-vessel disease (n=28)	1–19	3–10	5.5
Three-vessel disease (n=59)	0–20	2–6	4

* Kruskal–Wallis test demonstrated a significant difference according to the severity of anxiety symptoms between these groups.

## Discussion

We found an inverse association between coronary stenosis and the severity of anxiety symptoms among patients undergoing coronary angiography. 

Similar to our study, Elias et al.^28^ found an inverse association between coronary artery occlusion and anxiety symptoms. In the absence of organic disease, individuals with high anxiety and somatic symptoms show normal angiographic findings.^29^^, ^^30^ In a study in Lewiston, United States, the regression model suggested that trait anxiety was inversely correlated with the extent of occlusion. The authors concluded that women with low anxiety might be referred for catheterization later in the course of their disease.^31^ However, Tennant and Langeluddecke^32^ found no association between angiographic findings and psychological status including anxiety levels. Another study also showed no association between the severity of CAD involvement and the anxiety score. Nevertheless, in that study, the highest anxiety symptoms were found in patients with slow coronary flow.^33^

The magnitude of the association between coronary artery involvement and anxiety symptoms has been previously studied using other diagnostic procedures. Channer et al.^23^ showed a correlation between anxiety/depression and exercise test findings in patients with chest pain. In line with our findings, the authors found a normal exercise test to be more prevalent in patients with anxiety and depression.^23^ In another study, compared to patients who had CAD, individuals with normal angiography were found to have higher obsessive-compulsive traits, somatization, interpersonal sensitivity, phobic anxiety, paranoid ideation, and psychoticism.^34^^, ^^35^

Our result with respect to an inverse association between the extent of coronary stenosis in coronary angiography and psychological status warrants further research. It has been reported that patients with normal angiography may have higher rates of panic attacks and major depression disorder.^36^ Psychological distress, as well as psychiatric disorders, may contribute to how individuals experience and report somatic symptoms; this may increase the request for angiography and other evaluation procedures by physicians.^37^ It has been hypothesized that untreated anxiety among patients may exaggerate somatic presentations and patients may have a tendency to attribute their symptoms to their heart, rendering cardiologists more likely to request or perform angiography.^13^ These somatic symptoms can also influence how and when family physicians and general practitioners request cardiology consultation, which possibly results in angiographic evaluation and diagnosis of CAD in earlier stages. Autonomic nervous system disorders and hyperventilation due to anxiety mimic coronary angina while there is no or minimal evidence of coronary stenosis in angiography.^13^^, ^^29^ In a study, the patients’ anxiety level was found to influence the physician’s decision to request angiography and the authors concluded that angiography would reveal minimal findings in such cases.^36^

A high percentage of patients with anxiety who develop chest pain have normal angiographic findings. In the case of chest pain and anxiety, patients need a more precise screening of anxiety in addition to angiography. Validated and standardized instruments are available for administration in clinical practice for the screening of anxiety.^38^ There is a need for clinical trials to test the effects of diagnosis and anxiety alleviation in patients with comorbid anxiety and CAD, even if anxiety is inversely associated with the degree of coronary stenosis found in coronary angiography.^36^

The current study has the following limitations. First, the small sample size is a limitation. Second, we did not measure comorbid conditions in this study. Third, we measured symptoms of anxiety instead of the clinical diagnosis of any anxiety disorder. The cross-sectional design of the study is another drawback of note insofar as it did not permit any causal interference. Additionally, although among patients undergoing coronary angiography, 15% to 25% could have normal angiography, we did not include patients with normal coronary angiograms in the current study. Finally, we used the vessel score while there are more accepted scoring systems for assessing the extent and severity of CAD such as the Gensini score and the SYNTAX score.^39^^-^^41^

Our study also has clinical implications. This study reports an inverse relationship between anxiety symptoms and coronary stenosis, highlighting the need for more attention to the level of anxiety in patients who are single-vessel rather than 2- or 3-vessel. In other words, as patients with minimal angiographic findings have more anxiety, anxiety should be screened among patients in the early stages of CAD. Patients who display less impairment on an angiogram may benefit from the assessment and treatment of anxiety. This finding is important due to the prevalent prognostic role of anxiety symptoms in patients with CAD.^42^^-^^56^

## Conclusion

 Patients who undergo coronary angiography and have minimal coronary stenosis may have high levels of anxiety symptoms. The results advocate for anxiety screening and treatment among patients with coronary artery disease who have minimal angiographic findings. 

## Notes:

***This paper should be cited as: ***Assari S, Zandi H, Ahmadi K, Kazemi Saleh D. Extent of Coronary Stenosis and Anxiety Symptoms among Patients Undergoing Coronary Angiography. J Teh Univ Heart Ctr 2017;12(4):155-159.

## References

[1] Edéll-Gustaffson UM (2002). Insufficient sleep, cognitive anxiety and health transition in men with coronary artery disease: a self-report and polysomnographic study. J Adv Nurs.

[2] Edéll-Gustafsson UM, Hetta JE (1999). Anxiety, depression and sleep in male patients undergoing coronary artery bypass surgery. Scand J Caring Sci.

[3] Paterniti S, Zureik M, Ducimetière P, Touboul PJ, Fève JM, Alpérovitch A (2001). Sustained anxiety and 4-year progression of carotid atherosclerosis. Arterioscler Thromb Vasc Biol.

[4] Eaker ED, Sullivan LM, Kelly-Hayes M, D'Agostino RB Sr, Benjamin EJ (2005). Tension and anxiety and the prediction of the 10-year incidence of coronary heart disease, atrial fibrillation, and total mortality: the Framingham Offspring Study. Psychosom Med.

[5] Eaker ED (1989). Psychosocial factors in the epidemiology of coronary heart disease in women. Psychiatr Clin North Am.

[6] Hallman T, Burell G, Setterlind S, Odén A, Lisspers J (2001). Psychosocial risk factors for coronary heart disease, their importance compared with other risk factors and gender differences in sensitivity. J Cardiovasc Risk.

[7] Linfante AH, Allan R, Smith SC Jr, Mosca L (2003). Psychosocial factors predict coronary heart disease, but what predicts psychosocial risk in women. J Am Med Womens Assoc (1972).

[8] Sullivan MD, LaCroix AZ, Spertus JA, Hecht J (2000). Five-year prospective study of the effects of anxiety and depression in patients with coronary artery disease. Am J Cardiol.

[9] Thurston RC, Kubzansky LD (2007). Multiple sources of psychosocial disadvantage and risk of coronary heart disease. Psychosom Med.

[10] McCaffrey R, Taylor N (2005). Effective anxiety treatment prior to diagnostic cardiac catheterization. Holist Nurs Pract.

[11] Koivula M, Paunonen-Ilmonen M, Tarkka MT, Tarkka M, Laippala P (2001). Fear and anxiety in patients awaiting coronary artery bypass grafting. Heart Lung.

[12] Kornitzer M, Magotteau V, Degre C, Kittel F, Struyven J, van Thiel E (1982). Angiographic findings and the type A pattern assessed by means of the Bortner Scale. J Behav Med.

[13] Katon W, Hall ML, Russo J, Cormier L, Hollifield M, Vitaliano PP, Beitman BD (1988). Chest pain: relationship of psychiatric illness to coronary arteriographic results. Am J Med.

[14] Shibeshi WA, Young-Xu Y, Blatt CM (2007). Anxiety worsens prognosis in patients with coronary artery disease. J Am Coll Cardiol.

[15] Frasure-Smith N, Lespérance F (2008). Depression and anxiety as predictors of 2-year cardiac events in patients with stable coronary artery disease. Arch Gen Psychiatry.

[16] Lane D, Carroll D, Ring C, Beevers DG, Lip GY (2000). Do depression and anxiety predict recurrent coronary events 12 months after myocardial infarction?. QJM.

[17] Székely A, Balog P, Benkö E, Breuer T, Székely J, Kertai MD, Horkay F, Kopp MS, Thayer JF (2007). Anxiety predicts mortality and morbidity after coronary artery and valve surgery--a 4-year follow-up study. Psychosom Med.

[18] Shen BJ, Avivi YE, Todaro JF, Spiro A 3rd, Laurenceau JP, Ward KD, Niaura R (2008). Anxiety characteristics independently and prospectively predict myocardial infarction in men the unique contribution of anxiety among psychologic factors. J Am Coll Cardiol.

[19] Kuper H, Marmot M, Hemingway H (2002). Systematic review of prospective cohort studies of psychosocial factors in the etiology and prognosis of coronary heart disease. Semin Vasc Med.

[20] Katon W, Lin EH, Kroenke K (2007). The association of depression and anxiety with medical symptom burden in patients with chronic medical illness. Gen Hosp Psychiatry.

[21] Nekouei ZK, Yousefy A, Manshaee G, Nikneshan S (2011). Comparing anxiety in cardiac patients candidate for angiography with normal population. ARYA Atheroscler.

[22] Proudfit WL, Bruschke VG, Sones FM Jr (1980). Clinical course of patients with normal or slightly or moderately abnormal coronary arteriograms: 10-year follow-up of 521 patients. Circulation.

[23] Channer KS, Papouchado M, James MA, Rees JR (1985). Anxiety and depression in patients with chest pain referred for exercise testing. Lancet.

[24] Zigmond AS, Snaith RP (1983). The hospital anxiety and depression scale. Acta Psychiatr Scand.

[25] Bjelland I, Dahl AA, Haug TT, Neckelmann D (2002). The validity of the Hospital Anxiety and Depression Scale. An updated literature review. J Psychosom Res.

[26] Kudielka BM, von Känel R, Gander ML, Fischer JE (2004). The interrelationship of psychosocial risk factors for coronary artery disease in a working population: do we measure distinct or overlapping psychological concepts?. Behav Med.

[27] Montazeri A, Vahdaninia M, Ebrahimi M, Jarvandi S (2003). The Hospital Anxiety and Depression Scale (HADS): translation and validation study of the Iranian version. Health Qual Life Outcomes.

[28] Elias MF, Robbins MA, Blow FC, Rice AP, Edgecomb JL (1982). A behavioral study of middle-aged chest pain patients: Physical symptom reporting, anxiety, and depression. Experimental Aging Research.

[29] Herrmann C, Buss U, Breuker A, Gonska BD, Kreuzer H (1994). Relation of cardiologic findings and standardized psychological scales to clinical symptoms in 3,705 ergometrically studied patients. Z Kardiol.

[30] Kemp HG, Jr, Vokonas PS, Cohn PF, Gorlin R (1973). The anginal syndrome associated with normal coronary arteriograms. Report of a six year experience. Am J Med.

[31] Low KG, Fleisher C, Colman R, Dionne A, Casey G, Legendre S (1998). Psychosocial variables, age, and angiographically-determined coronary artery disease in women. Ann Behav Med.

[32] Tennant CC, Langeluddecke PM (1985). Psychological correlates of coronary heart disease. Psychol Med.

[33] Vural M, Satiroglu O, Akbas B, Goksel I, Karabay O (2009). Coronary artery disease in association with depression or anxiety among patients undergoing angiography to investigate chest pain. Tex Heart Inst J.

[34] Qiu YG, Zheng LR, Chen JZ, Zhu JH, Zhang FR, Xu Y, Zhao LL, Tao QM (2003). Psychologic status and their influencing factors in patients suspected of coronary disease before and after coronary catheterization. Zhonghua Liu Xing Bing Xue Za Zhi.

[35] Vural M, Satiroğlu O, Akbaş B, Göksel I, Karabay O (2007). Association between depression and anxiety symptoms and major atherosclerosis risk factors in patients with chest pain. Tohoku J Exp Med.

[36] Costa PT, Zonderman AB, Engel BT, Baile WF, Brimlow DL, Brinker J (1985). The relation of chest pain symptoms to angiographic findings of coronary artery stenosis and neuroticism. Psychosom Med.

[37] Waxler EB, Kimbiris D, Dreifus LS (1971). The fate of women with normal coronary arteriograms and chest pain resembling angina pectoris. Am J Cardiol.

[38] Young QR, Ignaszewski A, Fofonoff D, Kaan A (2007). Brief screen to identify 5 of the most common forms of psychosocial distress in cardiac patients: validation of the screening tool for psychological distress. J Cardiovasc Nurs.

[39] Yadav M, Palmerini T, Caixeta A, Madhavan MV, Sanidas E, Kirtane AJ, Stone GW, Généreux P (2013). Prediction of coronary risk by SYNTAX and derived scores: synergy between percutaneous coronary intervention with taxus and cardiac surgery. J Am Coll Cardiol.

[40] Capodanno D, Caggegi A, Miano M, Cincotta G, Dipasqua F, Giacchi G, Capranzano P, Ussia G, Di Salvo ME, La Manna A, Tamburino C (2011). Global risk classification and clinical SYNTAX (synergy between percutaneous coronary intervention with TAXUS and cardiac surgery) score in patients undergoing percutaneous or surgical left main revascularization. JACC Cardiovasc Interv.

[41] Chen SL, Han YL, Zhang YJ, Ye F, Liu HW, Zhang JJ, Xu B, Jiang TM, Zhou YJ, Lv SZ (2013). The anatomic- and clinical-based NERS (new risk stratification) score II to predict clinical outcomes after stenting unprotected left main coronary artery disease: results from a multicenter, prospective, registry study. JACC Cardiovasc Interv.

[42] Eng HS, Yean LC, Das S, Letchmi S, Yee KS, Bakar RA, Hung J, Choy CY (2011). Anxiety and depression in patients with coronary heart disease: a study in a tertiary hospital. Iran J Med Sci.

[43] Vural M, Satiroglu O, Akbas B, Goksel I, Karabay O (2009). Coronary artery disease in association with depression or anxiety among patients undergoing angiography to investigate chest pain. Tex Heart Inst J.

[44] Celano CM, Millstein RA, Bedoya CA, Healy BC, Roest AM, Huffman JC (2015). Association between anxiety and mortality in patients with coronary artery disease: A meta-analysis. Am Heart J.

[45] Saleh DK, Nouhi S, Zandi H, Lankarani MM, Assari S, Pishgou B (2008). The quality of sleep in coronary artery disease patients with and without anxiety and depressive symptoms. Indian Heart J.

[46] Assari S (2014). Intercourse avoidance among women with coronary artery disease. J Sex Med.

[47] Kazemi-Saleh D, Pishgou B, Assari S, Tavallaii SA (2007). Fear of sexual intercourse in patients with coronary artery disease: a pilot study of associated morbidity. J Sex Med.

[48] Kazemi-Saleh D, Pishgoo B, Farrokhi F, Fotros A, Assari S (2008). Sexual function and psychological status among males and females with ischemic heart disease. J Sex Med.

[49] Watkins DC, Assari S, Johnson-Lawrence V (2015). Race and ethnic group differences in comorbid major depressive disorder, generalized anxiety disorder, and chronic medical conditions. J Racial Ethn Health Disparities.

[50] Assari S, Moghani Lankarani M, Ahmadi K (2013). Comorbidity influences multiple aspects of well-being of patients with ischemic heart disease. Int Cardiovasc Res J.

[51] Bayat N, Alishiri GH, Salimzadeh A, Izadi M, Saleh DK, Lankarani MM, Assari S (2011). Symptoms of anxiety and depression: a comparison among patients with different chronic conditions. J Res Med Sci.

[52] Assari S (2016). Race and ethnic differences in additive and multiplicative effects of depression and anxiety on cardiovascular risk. Int J Prev Med.

[53] Vural M, Acer M, Akbaş B (2008). The scores of hamilton depression, anxiety, and panic agoraphobia rating scales in patients with acute coronary syndrome. Anadolu Kardiyol Derg.

[54] Bunevicius A, Staniute M, Brozaitiene J, Pop VJ, Neverauskas J, Bunevicius R (2013). Screening for anxiety disorders in patients with coronary artery disease. Health Qual Life Outcomes.

[55] Moryś JM, Bellwon J, Adamczyk K, Gruchała M (2016). Depression and anxiety in patients with coronary artery disease, measured by means of self-report measures and clinician-rated instrument. Kardiol Pol.

[56] Kim HY (2014). Analysis of variance (ANOVA) comparing means of more than two groups. Restor Dent Endod.

